# Clam Genome and Transcriptomes Provide Insights into Molecular Basis of Morphological Novelties and Adaptations in Mollusks

**DOI:** 10.3390/biology13110870

**Published:** 2024-10-25

**Authors:** Xiujun Sun, Xi Chen, Biao Wu, Liqing Zhou, Yancui Chen, Sichen Zheng, Songlin Wang, Zhihong Liu

**Affiliations:** 1 State Key Laboratory of Mariculture Biobreeding and Sustainable Goods, Yellow Sea Fisheries Research Institute, Chinese Academy of Fishery Sciences, Qingdao 266071, China; xjsun@ysfri.ac.cn (X.S.); chenxi2647150@163.com (X.C.); wubiao@ysfri.ac.cn (B.W.); zhoulq@ysfri.ac.cn (L.Z.); zhengsichen1019@163.com (S.Z.); wslin1126@163.com (S.W.); 2 Laboratory for Marine Fisheries Science and Food Production Processes, Laoshan Laboratory, Qingdao 266071, China; 3 Zhangzhou Aquatic Technology Promotion Station, Zhangzhou 363000, China; 15260121656@163.com; 4 College of Fisheries and Life Science, Shanghai Ocean University, Shanghai 201306, China; 5 College of Marine Science and Fisheries, Jiangsu Ocean University, Lianyungang 222005, China

**Keywords:** clam, genome, transcriptome, phenotype evolution, transposable elements

## Abstract

Bivalve mollusks, comprising animals enclosed in two shell valves, are well-adapted to benthic life in many intertidal zones. Clams have evolved the buried lifestyle, which depends on their unique soft tissue structure and their wedge-shaped muscular foot and long extendible siphons. However, molecular mechanisms of adaptative phenotype evolution remain largely unknown. In the present study, we obtain the high-quality chromosome-level genome of Manila clam *R. philippinarum*, an economically important marine bivalve in many coastal areas. The genome is constructed by the Hi-C assisted assembly, which yields 19 chromosomes with a total of 1.17 Gb and BUSCO integrity of 92.23%. The de novo assembled genome has a contig N50 length of 307.7 kb and scaffold N50 of 59.5 Mb. Gene family expansion analysis reveals that a total of 24 single-copy gene families have undergone the significant expansion or contraction, including E3 ubiquitin ligase and dynein heavy chain. The significant expansion of transposable elements has been also identified, including long terminal repeats (LTR) and non-LTR retrotransposons. The comparative transcriptomics among different clam tissues reveals that extracellular matrix (ECM) receptors and neuroactive ligand receptors may play the important roles in tissue structural support and neurotransmission during their infaunal life. These findings of gene family expansion and tissue-specific expression may reflect the unique soft tissue structure of clams, suggesting the evolution of lineage-specific morphological novelties. The high-quality genome and transcriptome data of *R. philippinarum* will not only facilitate the genetic studies on clams but will also provide valuable information on morphological novelties in mollusks.

## Simple Summary

Clams have evolved the buried lifestyle, which depends on their unique soft tissue structure and their wedge-shaped muscular foot and long extendible siphons. However, the molecular mechanism of adaptative phenotype evolution remains largely unknown. The analysis of genomic data showed significant expansion or contraction in specific gene families and transposable elements. Comparative transcriptomics between different tissues reveals that extracellular matrix (ECM) receptors and neuroactive ligand receptors may play important roles in tissue structural support and neurotransmission in clams. The high-quality genome and transcriptome of *Ruditapes philippinarum* will provide valuable information on morphological novelties in mollusks.

## 1. Introduction

Recent advancements in cost-effective genome sequencing have greatly improved our understanding of molluscan biology and evolution, especially in the “evo-devo” field [1,2,3,4,5,6]. An intriguing hypothesis is that the vast diversity of molluscan morphology and evolution is probably due to the lack of skeletal constraints, supported by gene family expansion and widespread staggered expression, known as a spatial non-collinear expression pattern [7,8,9,10,11,12,13]. How the diverse morphological patterns and lifestyles of mollusks have evolved is still largely unknown and hampered by limited molecular information. 

Mollusks are one of the most diverse and evolutionarily successful groups in the animal kingdom, including over 100,000 described extant species [1,2,3]. Distributed widely in marine, freshwater, and terrestrial ecosystems, they display tremendous diversity of morphology and lifestyle [4,5]. Mollusks have considerable economic and ecological significance, serving as important seafood sources for humans [4,6]. Bivalve mollusks (e.g., clams, oysters, mussels, and scallops) comprise animals enclosed in two shell valves, playing important roles in many intertidal zones [1,14]. Many bivalves are important fishery and aquaculture species, providing significant economic benefits to humans, with the global production of almost 17 million tons from aquaculture [15,16]. Bivalve aquaculture has been expanded worldwide and achieved its commercial success, mainly owing to a global appreciation of the soft body meat with delicious taste [4,8,17,18]. Although bivalves usually have similar shell phenotypes, their body sizes are incredibly variable, ranging from the small dwarf clam *Mulinia lateralis* (shell length of 15–20 mm) to the giant clam *Tridacna gigas* (shell length of 1.3–1.8 m) [19]. Bivalves have also evolved different soft tissue structure adapted to different lifestyles, such as buried, sessile, or attached filter feeders, suggesting morphological novelties and habitat adaptations in marine ecosystems [1,4,6,8]. 

Clams are a very diverse group of bivalves with notable variation in shape, size, color and body structure [5]. They represent an important group of burrowing animals that have to cope with highly variable salinity, temperature, and dissolved oxygen levels in estuarine and intertidal regions [1,20,21]. The burrowing behavior of clams has been proved to be associated with their unique body structures, e.g., a wedge-shaped muscular foot and long extendible siphons [1,22,23,24]. The burrowing ability allows clams to penetrate sand with the posterior foot and maintain water-exchange above the sediments by the inhalant and exhalant siphons [1]. Although neural control of body locomotion is conserved from mollusk to man, the essential molecular components for the neural control responsible for clam burrowing remain largely unknown [2,23,24,25,26,27]. Recent work on scallop muscles has revealed a diverse repertoire of neurons in molluscan muscle tissues, suggesting that scallop swimming may be regulated by motor ganglia [7,20,26]. However, the molecular basis of these morphological novelties in clams are still largely unknown.

In the present study, we investigate a chromosome-level assembled genome for the Manila clam *Ruditapes philippinarum*, an economically important marine bivalves living naturally under sediments in many coastal areas of China [18,20]. In recent years, *R. philippinarum* has become one of the major cultured shellfish across the world, mainly due to its good flavor and affordable prices [18,20,28]. According to the previously assembled genomes, molecular signatures of tolerance to variable environmental conditions have been investigated in the clam *R. philippinarum* [18,19,20,21,29,30,31]. Despite this, the molecular basis of morphological novelties (e.g., a wedge-shaped muscular foot and long extendible siphons) is still largely unknown. In this study, the chromosome-level genome and tissue transcriptomes of *R. philippinarum* will not only provide unprecedented insights into the molecular basis of the unique morphological features in clams but also enhance our understanding of molluscan biology and evolution.

## 2. Materials and Methods

### 2.1. DNA Extraction and Sequencing Library Preparation

The healthy clams of *R. philippinarum* (average shell length: 37.68 ± 0.68 mm) were collected from the coastal area of Chaozhou (China). The clams were cultured in seawater for one week at the Yellow Sea Fisheries Research Institute (YSFRI) before the sampling. Fresh tissues of the clams were collected and immediately frozen in liquid nitrogen. All samples were stored at −80 °C for DNA and mRNA extraction. Genomic DNA was extracted from the foot tissue using a DNA/RNA Extraction Kit (Vazyme, Nanjing, China). The quality of genomic DNA was assessed by the Qubit Fluorometric system and gel electrophoresis system. DNA fragments greater than 20 kb in length were selected for preparation of the sequencing library using a BluePippin instrument (Sage Science, Beverly, MA, USA). The PacBio library was constructed using SMRTbell Template Prep Kit-SPv3 following the manufacturer’s recommendations. The quality control of the obtained library was performed using Qubit and Agilent 2100. The library preparation was mainly divided into the following five steps: (1) genomic DNA shearing; (2) DNA damage repair and A-tailing; (3) ligation with hairpin adapters; (4) nuclease treatment of SMRTbell library; (5) size selection and binding to the polymerase. Briefly, 15 µg genomic DNA was sheared with gTUBEs to generate fragments of more than 8 Kb. The single-strand overhangs were then removed, and DNA fragments were used for damage repair, end repair, and A-tailed. Subsequently, the fragments were ligated with the hairpin adapters for PacBio sequencing. The library was treated with the nuclease using the SMRTbell Enzyme Cleanup Kit, purified by AMPure PB Beads, and screened by BluePippin (Sage). The final library was sequenced on the Pacific Biosciences Sequel system (Genedenovo company, Guangzhou, China) to generate long sequencing reads.

### 2.2. Genome Survey

Genomic DNA was sheared to an average size of 500 bp, with randomly fragmented inserts ranging from 300 to 700 bp. The quality of genomic DNA was assessed by the Qubit Fluorometric system and gel electrophoresis. An Illumina sequencing library was prepared with an insert size of 500 bp using a Paired-End DNA Sample Prep kit (Illumina Inc.; San Diego, CA, USA). They were sequenced using novaseq6000 (Genedenovo company, Guangzhou, China) to produce short reads. Clean reads were obtained after filtering and correction of the raw sequencing data. The size of the genome, repetitive sequences, and heterozygosity were estimated by using jellyfish (version 2.2.6) and GenomeScope (version 1.0.0) [32].

### 2.3. Genome Sequencing and Assembly

The *R. philippinarum* genome was sequenced by PacBio third-generation and Illumina second-generation sequencing technology combined with HiC technologies to obtain the chromosome-level genome. Sequencing was performed on a PacBio Sequel II instrument with Sequencing Primer V2 and Sequel II Binding Kit 2.0 at the Genome Center of Grandomics. Raw reads were processed to get high quality of clean reads according to the following filtering conditions: (1) remove reads with more than 10% unidentified nucleotides; (2) remove reads with more than 50% bases having quality scores of less than 20; (3) remove reads mapped to the barcode adapters.

The accuracy was determined by the realignment of short reads to the assembled genome. The mapping rate, genome coverage and depth distribution were calculated for the newly assembled genome. EST (Expressed Sequence Tag) sequences from *R. philippinarum* were aligned to the genome assembly by BLAST 2.2.29 to evaluate the integrity of the genome assembly by means of CEGMA v2.5 (Core Eukaryotic Genes Mapping Approach) [33] and BUSCO version 3.0 (Benchmarking Universal Single-Copy Orthologs) [34]. The reference BUSCO databases were set as metazoa_odb9. Genome assembly was completed by the software MECAT v1.0 to assemble the third-generation sequencing reads, and then the software Pilon v1.23 to align the second-generation sequencing reads to the assembled genome sequence. The genome results were corrected according to the default parameters. The GC content and average sequencing depth were counted using 500 bp non-overlapping sliding windows.

### 2.4. Genome Annotation

Genome annotation was mainly performed using repeat sequence annotation, coding genes annotation and non-coding RNA (ncRNA) annotation. Non-coding RNA (e.g., rRNA, tRNA, snRNA) were predicted by RNAmmer, tRNAscan-SE 2.0 and Rfam database V13 [35,36]. Tandem repeats finder and RepeatMasker v4.05 were used to predict the tandem repeats in the newly assembled genome [37,38,39]. Due to their specific sequence characteristics, repeat sequences were predicted by different methods: (1) LTR transposons were predicted through LTR_FINDER v1.07 [39]; (2) Helitron transposons were predicted by Helitroscanner v1.1; (3) MITE transposons were predicted by MITE-Hunter [40]; and (4) LINE transposons were predicted by MGEscan-nonLTR v1.1 [41]. Furthermore, repeat sequences were also classified according to the construction of the de novo method. The preliminary de novo prediction results were obtained by PILER v1.0, RepeatScout v1.05 and RepeatModeler v2.0.1 [42,43,44]. The constructed repeat sequence database was integrated with the Repbase database as the final repeat sequence database, which was used for repeat sequence prediction by RepeatMasker v4.05 [44]. 

For gene annotation of the assembled genome, Augustus and GeneMark were used to predict the coding genes according to Hidden Markov Model (HMM) [45,46]. The homology prediction was performed by MAKER-P v2.29 [47]. RNA-seq data was integrated through hisat2 and StringTie to obtain a gene set predicted by the transcriptome [48,49]. To obtain the final gene sets, MAKER-P v2.29 was used to integrate the obtained RNA-seq data, homologous results, and de novo prediction. The homologous genes were defined according to a two-way alignment, with the alignment threshold at e-value < 1 × 10^−5^ and query cov > 30%. Functional annotation of coding genes is mainly based on the functionally homologous sequences in the annotated databases, including non-redundant protein database (NR), Gene Ontology (GO), SwissProt, Kyoto Encyclopedia of Genes and Genomes (KEGG) and Clusters of Orthologous Groups (COG). The predicted protein sequences were aligned with protein function databases using BLAST 2.2.29 by threshold value of 1 × 10^−5^ [50]. 

### 2.5. Genome Evolution and Gene Family Expansion Analysis

The coding genes of *R. philippinarum* and 12 other invertebrate species were selected for comparative genomic analysis, including seven bivalves (*Mytilus coruscus*, *Patinopecten yessoensis*, *Crassostrea gigas*, *Cyclina sinensis*, *Sinonovacula constricta*, *Pinctada fucata martensii* and *Pecten maximus*), three gastropods (*Lottia gigantea*, *Pomacea canaliculata* and *Aplysia californica*), one cephalopod (*Octopus sinensis*) and one polychaeta (*Capitella teleta*) as the outgroup [9,11,51,52,53,54,55,56,57,58].

The orthologous genes among species were identified by Diamond and OrthoMCL by default parameters with *E*-value < 1 × 10^−5^ and query coverage > 30% [59,60]. The GO function annotation and KEGG enrichment analysis were then conducted for the obtained genes. According to protein sequences of single-copy orthologous genes, the phylogenetic tree was constructed according to the following steps. First, the different protein sequences were aligned to identify the single-copy orthologous gene family in different species using MUSCLE v3.8.31 [43]. The results of multiple sequence alignment of proteins were then converted into multiple sequence alignment of nucleic acids by using protein sequences and their corresponding nucleic acid sequences. The multiple sequence alignment results of all single-copy gene family of nucleic acid sequences were spliced together end-to-end to obtain the total single-copy gene sequence file, which was used for the tree construction. The divergence time was estimated using TimeTree 5 (http://www.timetree.org/, accessed on 14 September 2021) and MCMCtree in PAML v4.10.6, with six calibration points according to the previous studies [61]. Finally, the phylogenetic tree was constructed by RAxML-III v1.2.0 using the maximum likelihood method [62,63]. 

Molluscan species were selected for gene family analysis according to their different lifestyles, including infaunal type, attached type, semi-sessile type, semi-sessile type, sessile type, semi-buried type, and swimming type. According to the genome data availability, six molluscan genomes were selected for gene family analysis, including *R. philippinarum* (Rph; infaunal type), *P. yessoensis* (Pye; attached type), *Chlamys farreri* (Cfa; semi-sessile type), *C. gigas* (Cgi; sessile type), *Scapharca broughtonii* (Sbr; semi-buried type), and *Octopus bimaculoides* (Obi; swimming type), according to the previously assembled genomes [7,27,51,64]. The analysis for gene family expansion and contraction in *R. philippinarum* was calculated according to the most recent common ancestor inferred with CAFÉ v2.1 using a random birth and death process model [65]. The criteria for defining significant expansion or contraction of gene families were set as *p*-values less than 0.05. The expanded gene families were also used for the Kyoto Encyclopedia of Genes and Genomes (KEGG) and Gene Ontology (GO) enrichment analyses to obtain their functional annotation results. Fisher’s exact test was used to identify the significantly enriched GO and KEGG pathways among the expanded and contracted gene families, followed by a false discovery rate correction (FDR < 0.05). Furthermore, the genome of hard clam *Mercenaria mercenaria* [66] was selected for analysis of gene family expansion and contraction in order to test the potential convergent evolution of the infaunal bivalves (e.g., *R. philippinarum* and *Mercenaria mercenaria*).

### 2.6. Comparative Transcriptomics Among Tissues and WGCNA

Total RNA was extracted from different issues of clams (foot, siphon, mantle, gill, gonad, adductor muscle and hepatopancreas) by the traditional Trizol method (Invitrogen, San Francisco, CA, USA) according to the manufacturer’s instruction. Three replicates were used for each tissue. The purity and quality of RNA were estimated by NanoPhotometer spectrophotometer (Implen, Los Angeles, CA, USA) and 1.0% agarose electrophoresis. Library construction for transcriptome sequencing was performed according to our previous study [20]. The library was estimated by size selection, using polymerase chain reaction (PCR) and gel electrophoresis, and subsequently used for next-generation sequencing on Illumina HiSeqTM 4000 platform. Transcriptome data from different tissues can not only be used for the genome assembly and annotation, but also for comparative transcriptome analysis to reveal differentially expressed genes among tissues. The raw reads were filtered by fastp version 0.18.0, using the following parameters: (1) removing the reads having adapters; (2) removing the reads with more than 10% of unknown nucleotides; and (3) removing low-quality reads with more than 50% of low-quality bases. Bowtie2 (version 2.2.8) was used for short reads alignment by mapping reads with ribosome RNA (rRNA) database. After removing rRNA mapped reads, the remaining clean reads were further used in assembly and gene abundance calculation. An index of the reference genome was built, and paired-end clean reads were mapped to the reference genome using HISAT2 2.1.0. The mapped reads were further assembled by using the reference-based approach in StringTie v1.3.1. For each transcription region, a FPKM (fragment per kilobase of transcript per million mapped reads) value was calculated to quantify its expression abundance using RSEM software v1.3.3. The original reads count is not conducive to the comparison of differentially expressed genes (DEGs) among samples, while FPKM method can eliminate the potential effects of gene length and sequencing differences on gene expression. The data analysis for reads count was performed by DESeq2 V1.20.0, including normalization of reads count, and calculation of false positive rates (FDR) [67]. The calculation of FPKM values was used to obtain the fold changes of reads count. In the present study, DEGs among tissues were screened at the criteria of FDR < 0.05 and |log_2_Foldchanges| > 1 [67]. The heatmap was constructed according to the normalized z-score FPKM values. The heatmap was made by hierarchical cluster analysis, using the pheatmap package in R with a corrected *p* < 0.05 as the significance threshold. Differentially expressed genes were selected for functional annotation by GO and KEGG pathways. The relative expression levels of non-LTR transposable elements, dynein heavy chain (DHC), ECM components (e.g., laminin and collagen), and neuroactive ligand were compared among different tissues to investigate the pattern of tissue-specific gene expression in the clams.

The WGCNA (weighted gene co-expression network analysis) was performed using different tissue transcriptomes [68]. The low-quality genes were screened by the following condition, gene expression levels greater than 0.1 in more than 70% of the samples. All the remaining genes were used for WGCNA analysis. Module eigengenes were used to calculate the correlation coefficient among samples to find out the significant modules. Module significance was determined as the average absolute gene significance across the module genes [68]. GO and KEGG enrichment analysis were further conducted for the functional annotation of these modules at a threshold of false discovery rate (FDR) less than 0.05.

### 2.7. Immunohistochemical (IHC) and Neuronal Staining

The clam tissues were dissected and fixed in 4% PFA for immunohistochemical staining. The paraffin sections of different tissues were prepared using a Leica RM2016 microtome. Immunohistological staining of laminin and collagen was performed, according to our previous study, with minor modifications [69]. Briefly, the tissue samples were cut into small pieces and fixed in the general tissue fixing agent (Servicebio, Wuhan, China). After dehydration with gradient ethanol and xylene, the samples were processed with the Leica RM2016 by routine sectioning. The antigen retrieval and overnight incubation were performed for those sections. After rinsing with PBST, the primary antibody of Collagen I rabbit polyclonal antibody (Servicebio, Wuhan, China) was used with overnight incubation at 4 °C. Sections were washed with PBS (PH 7.4) and incubated with HRP-conjugated secondary antibody (Servicebio, Wuhan, China) for 50 min at room temperature. Sections were rinsed three times with PBS to remove unbound antibodies. Treated sections were incubated in DAB solution and then in DAPI staining for cell nuclei. Paraffin sections were also used for classical Nissl staining to identify the presence and distribution of neurons in clam tissues, especially in the foot and in the siphons [70].

## 3. Results and Discussion

### 3.1. Genome Feature of Clam R. philippinarum

**Figure 1:**
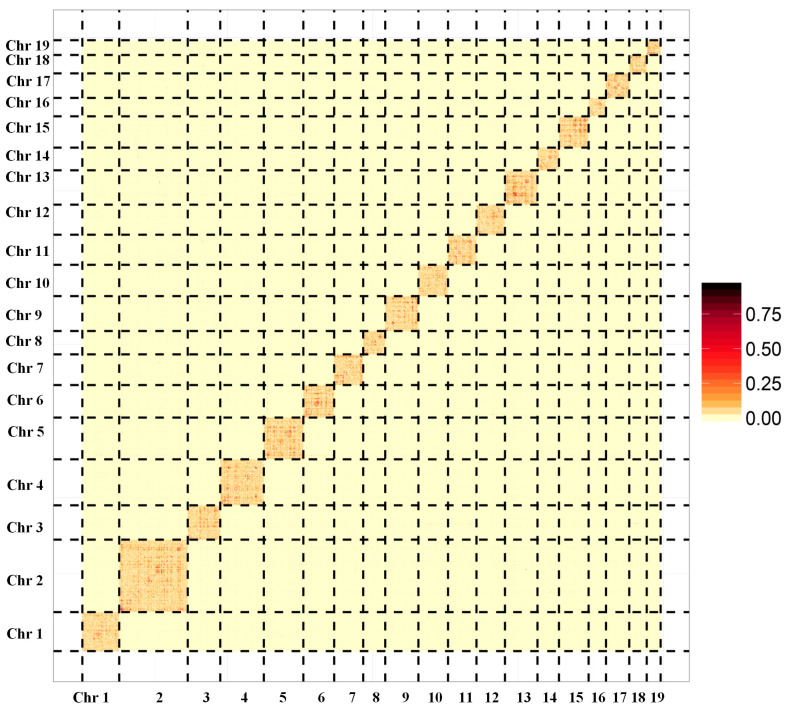
The genome-wide Hi-C map for Manila clam *Ruditapes philippinarum*. The names from Chr1 to Chr19 represent the 19 pseudochromosomes. The color blocks represent the correlation between one location and the other locations in the assembled genome.

**Figure 2:**
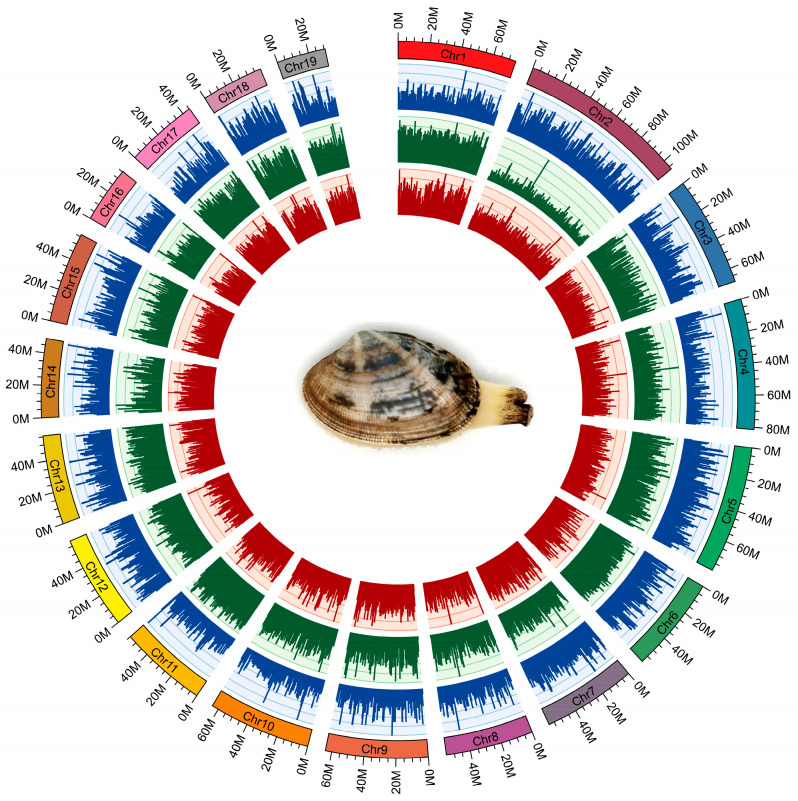
The circular genomic map for Manila clam *R. philippinarum*. From outer to inner circles: 19 chromosomes, gene density (blue), repeat density (green) and GC density (red) across the genome (a sliding window of 500 k). For the outer circle, the rectangular color bars represent different chromosomes in *R. philippinarum* genome.

Pacbio and Illumina sequencing were used for de novo assembly of the clam *R. philippinarum* genome. The sperm or eggs were collected from the gonads of clams for sex determination using a light microscope. One male clam with sperms was selected for genome survey sequencing to estimate genome size and heterozygosity by analyzing k-mer frequency. As a result, the genome size of *R. philippinarum* was estimated to be 1.17 Gb, showing a high heterozygosity of 3.30% and a moderate repeat rate of 52.02%. The PacBio sequencing produced a total of 108.9 Gb long reads, with an average length of ~10kb and an N50 of ~17.7 kb. After removing low-quality reads, the Illumina sequencing generated 412 million clean reads (99.34%), comprising 62.3 Gb. To evaluate the accuracy of genome assembly and sequencing, we compared Illumina reads to the raw assembled genome and found that 98.73% of reads mapped. The chromosome-level genome was constructed by Hi-C assisted assembly, yielding 19 chromosomes with a total of 1.17 Gb and BUSCO integrity of 92.23% (Figure 1). The assembled genome data files for *R. philippinarum* were provided in the Supplementary data. The de novo assembled genome had a contig N50 length of 307.7 kb and scaffold N50 of 59.5 Mb (Table 1). Features of the 19 chromosomes, including gene density, repeat density, and GC density across the genome, were illustrated in a circular genomic map for *R. philippinarum* (Figure 2). In this study, the assembled genome had a contig N50 length of 307.7 kb, which is higher than that in the recent long-read-based genome of *R. philippinarum* (contig N50 length of 182.7 kb) [31]. The contig N50 in this study is almost 48-fold and 10-fold higher than those in previous studies, with contig N50 sizes of 6.5 kb [29] and 28 kb [18], respectively. Thus, the high-quality, chromosome-level assembly of *R. philippinarum* presented here is a significant improvement over the previously published clam genomes. A total of 5371 scaffolds (covering 88.3% of the assembly) were assigned to the 19 chromosomes, which was consistent with the karyological characterization of *R. philippinarum* showing a diploid chromosome number of 2n = 38 [71]. The same diploid chromosome number was also found in other bivalves [7,9,11,53].

The predicted non-coding RNA (ncRNA), including rRNA, tRNA, snRNA, and miRNA, accounted for less than 0.05% of the clam genome (Supplementary Table S1). Repetitive sequences in the assembled genome were identified by de novo prediction and homology construction, which produced a total of ~637 Mb repetitive sequences, accounting for 52.02% of the clam genome (Supplementary Table S2; Supplementary Figure S1). The repeat contents in the genome of *R. philippinarum* were relatively higher than those in many mollusks (32.1–48.31%), including scallops, clams, oysters, mussels, and octopus [7,9,27,51,64,72]. In contrast, the highest repeat content in molluscan genomes was reported in shallow-water mussel *Modiolus philippinarum* (62.0%). Variation of repetitive sequences in molluscan genomes likely contributes to variation in their genome sizes [8]. 

As the major component of repetitive sequences in animal genomes, transposable elements (TEs; also known as “mobile DNAs”) are widely accepted as important drivers of genome architecture and evolution [19,73,74,75]. TEs can be classified mainly into two types: DNA transposons and retrotransposons [76]. According to the presence or absence of long terminal repeats (LTRs), retrotransposons are usually divided into LTR and non-LTR retrotransposons. In *R. philippinarum* genome, the most abundant members of TEs are detected in LTR retrotransposons (6071), nearly half of which are recognized as Gypsy (3022; Supplementary Figure S1; Supplementary Table S2). Like other animals, Gypsy elements are also recognized as the most abundant superfamily in molluscan genomes, appearing highly diversified families and clades [19,77]. As reported in *Drosophila*, the horizontal transfer events of Gypsy elements have undergone amplification and aberrations, leading to the rise of their diverse variants [78]. Despite this, a low proportion of LTR retrotransposons was found in the clam genome, accounting for only 3.25% of the genome size. In contrast, DNA transposons account for the highest proportion of TE classes (37.69%), the majority of which are recognized as Helitron. The rapid spread of Helitrons among animal lineages by horizontal transfer is probably facilitated by shuttling in viral particles mediated by close organism associations [79,80]. For non-LTR retrotransposons, the members are diversified with 34 superfamilies, most of which are derived from LINE elements (~84%; Supplementary Table S2), which are identified as being greatly contributed to the genomes of nonbony vertebrates and some actinopterygian fish [74]. In the present study, we reveal the specific pattern of transposable element diversity in *R. philippinarum* genome, displaying abundant members of LTR retrotransposons, high genomic proportion of DNA transposons, and diversified non-LTR retrotransposons. The impacts of transposable elements on genome evolution in mollusks are probably derived from generating insertion mutations, altering gene expression, and contributing to genetic innovation [19,76]. However, the functional consequences of TE diversity on molluscan genomes remain to be further investigated to uncover the events of loss and gain during evolution.

According to the prediction results from HMM (hidden Markov model), homologous alignment, and RNA-seq data, the assembled genome has 37,428 protein-coding genes, with an average gene length of 5,958 bp and an average CDS length of 1193 bp (Supplementary Table S2). Furthermore, PacBio sequencing data was used to improve the accuracy of gene prediction by long sequencing reads. The four databases (NR, SwissProt, GO, COG and KEGG) were used for gene functional annotation, indicating that most of the protein-coding genes (27,298) were annotated in the NR database, accounting for 73.0% (Supplementary Figure S2).

The completeness of BUSCO assessment for the protein-coding genes was calculated to be 89.1%, which is comparable to that of the recent genome of *R. philippinarum* (83.4%), hard clam *M. mercenaria* (90.5%), Peruvian scallop *Argopecten purpuratus* (89%), razor clam *S. constricta* (91.9%) [10,66,81]. According to the BUSCO assessment using the metazoan dataset, the completeness of molluscan genomes is reported to vary from 81.9% to 94.6%, which may be affected by the highly dynamic repeat contents and high heterozygosity in mollusks [5,9].

### 3.2. Comparative Analysis and Phylogeny of Molluscan Genomes

**Figure 3:**
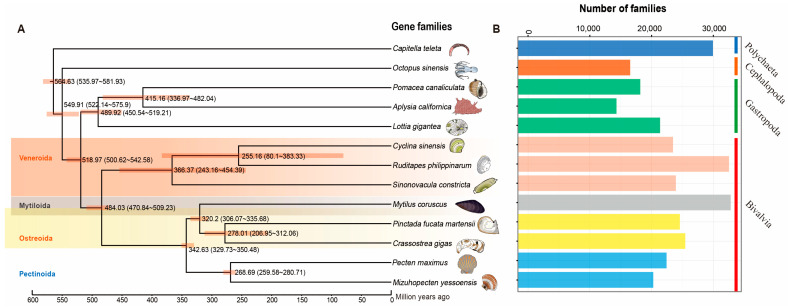
Phylogenetic analysis of *R. philippinarum* with other 12 invertebrate species. The polychaeta *Capitella teleta* was used as the outgroup. Estimated divergence times (million years ago) between lineages and 95% confidential intervals are labeled at each branch site. (**A**) The phylogenetic tree constructed by single-copy genes, showing divergence times (million years ago, Ma) and 95% confidence limits of divergence times in parentheses; (**B**) Comparison of the number of homologous gene families among species. The vertical axis represents different species, while the horizontal axis represents the number of gene families in each species.

A Venn diagram displayed common and unique gene families among the 13 invertebrates (Supplementary Figure S3). Only 1740 common gene families were detected among these species. A phylogenetic tree was constructed from protein-coding gene sequences from *R. philippinarum* and other 12 invertebrate species, including hard-shelled mussel (Mco: *M. coruscus*), Yesso scallop (Mye: *P. yessoensis*), Pacific oyster (Cgi: *C. gigas*), Venus clam (Csi: *C. sinensis*), Razor clam (Sco: *S. constricta*), Pearl oyster (Pma: *P. fucata martensii*), East Asian common octopus (Osi: *O. sinensis*), Limpet (Lgi: *L. gigantean*), Apple snail (Pca: *P. canaliculate*), Sea hare (Aca: *A. californica*), King scallop (Pma: *P. maximus*), and, as an outgroup, the marine polychaete (Cte: *C. teleta*). As expected, the eight bivalves (*R. philippinarum*, *C. sinensis*, *P. fucata martensii*, *S. constricta*, *M. coruscus*, *C. gigas*, *P. yessoensis* and *P. maximus*) were grouped together with three gastropods (*L. gigantea*, *P. canaliculata*, and *A. californica*) forming an independent branch. For bivalve mollusks, the most recent divergence time (~250 million years ago) was found in Veneridae, indicating the close relationship between *R. philippinarum* and *C. sinensis* (Figure 3A). The lineage of Veneridae and razor clam *S. constricta* were clustered into the clade of Veneroida, showing the divergence time of ~360 MYA (million years ago). The Veneroida diverged from other bivalves (Mytiloida, Ostreoida, and Pectinoda) at ~480 MYA (Figure 3A). The most abundant gene families (>3000) were found in the two bivalves, clam *R. philippinarum* and mussel *M. coruscus* (Figure 3B). The Bivalvia lineage diverged from Gastropoda at ~520 MYA (million years ago), indicating a sister-taxon relationship between Gastropoda and Bivalvia [7,82]. In contrast, cephalopods diverged from the ancestor lineage of Gastropoda and Bivalvia at ~550 MYA. This corresponds to the previous findings of Paleozoic (cephalopods) and Modern (bivalves) faunas [83]. It is therefore suggested that cephalopods may have evolved independently from Gastropoda and Bivalvia, probably due to the independent origins of complex brains and neurons in cephalopods [82,84,85]. The present study provides a high-quality genome of *R. philippinarum* with sufficient resolution to investigate evolutionary relationships among mollusks and help recover a well-supported topology for Mollusca. Clam species from Veneridae were most recognized as the borrowing bivalves, having the well-adapted morphology for an infaunal lifestyle [9,22,30]. The phylogenetic tree at the genome level supports the evolutionary relationships of mollusks, suggesting the genome dynamics and evolution in mollusks.

### 3.3. Gene Family Expansion and Contraction

**Figure 4:**
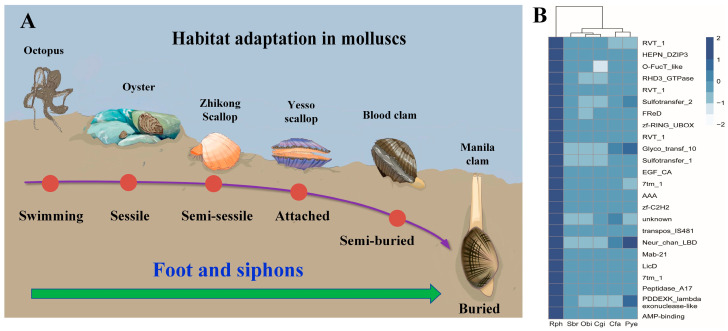
The habitat adaptation in mollusks. (**A**) the heatmap for the conserved domains of expanded gene families in six molluscan species with different lifestyles. Manila clam (*R. philippinarum*), Oyster (*C. gigas*), Yesso scallop (*P. yessoensis*), Zhikong scallop (*Chlamys farreri*), Blood clam (*Scapharca broughtonii*), and Octopus (*Octopus bimaculoides*). The selected gene families (z-score standardization) are displayed as y-axis, and x-axis represents the number of genes in each species in the corresponding gene family. The darker color represents the greater number of gene families, highlighting the significant expansion of *R. philippinarum*. (**B**) Rph (*R. philippinarum*), Cgi (*C. gigas*), Pye (*P. yessoensis*), Cfa (*C. farreri*), Sbr (*S. broughtonii*), and *Obi* (*O. bimaculoides*).

**Figure 5:**
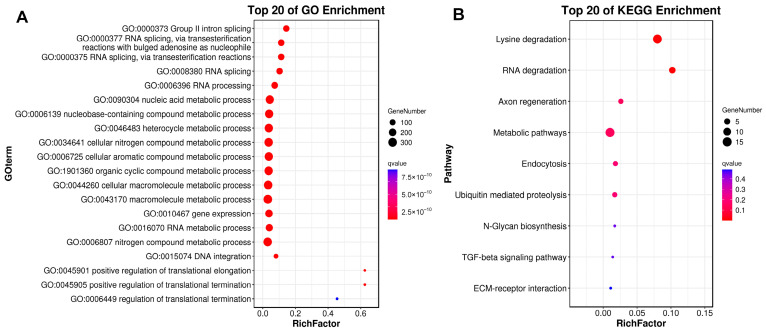
GO and KEGG enrichment results for expanded gene families. The horizontal axis of RichFactor represents the ratio of differential genes located in GO and KEGG.

As previously described, gene family expansion and contraction have been investigated according to the most recent common ancestor inferred with CAFÉ v2.1. Six molluscan species (*R. philippinarum*, *C. gigas*, *P. yessoensis*, *C. farreri*, *S. broughtonii* and *O. bimaculoides*), representing different lifestyles were used to identify expanded and contracted gene families (Figure 4). The habitat adaptation in mollusks is displayed in Figure 4A. The analysis of gene families indicated that there were 1408 gene family expansions and 12,176 gene family contractions identified in *R. philippinarum* genome. Among them, 24 single-copy gene families showing significant expansions or contractions were identified as the rapidly evolving families (*p* < 0.05; Figure 4B). The GO enrichment for the 24 gene families revealed that they were mainly involved in RNA splicing, RNA processing, metabolic process and DNA integration. The KEGG pathway analysis indicated that these expansion of gene families were mainly enriched in lysine degradation, axon regeneration, metabolic pathways, endocytosis, ubiquitin mediated proteolysis, N-glycan biosynthesis, TGF-beta signaling pathway, and ECM-receptor interaction (Figure 5). 

For the Manila clam, the 24 expanded gene families representing the conserved domains have been illustrated in the heatmap (Figure 4, Supplementary Figure S4). Comparisons among the six molluscan species revealed conserved domains in these expanded gene families, including RVT_1, HEPN_DZIP3, AAA, RHD3_GTPase, FReD, 7tm_1, Neu_chan_LBD, zf-C2H2, etc. Together with the hard clam *M. mercenaria*, we find that the infaunal bivalves (*R. philippinarum* and *M. mercenaria*) share some of the same gene family expansions, such as interferon-induced very large GTPase, mobile element jockey-like, E3 ubiquitin-protein ligase, neuronal acetylcholine receptor, and fibrinogen C domain-containing protein (Supplementary Table S4). According to GO and KEGG enrichment analysis, these expanded gene families are mainly involved in amino acid metabolism and neuroregulation (Supplementary Figure S5). It is therefore suggested that the infaunal bivalves may have evolved their unique metabolic mechanism and neuroregulatory system for habitat adaptation, which may arise independently in different lineages (i.e., convergent evolution) [86,87]. 

Notably, a significant expansion of transposable elements (TE) was also found in the Manila clam, including non-LTR retrotransposon (*jockey-like*, *jockey*, and *pol-like*), DNA transposons (*IS481*), and LTR retrotransposon (*pao/BEL*) (Supplementary Table S3). Three elements (*pol-like*, *IS481*, *pao/BEL*) were detected in *R. philippinarum* but not in the other five molluscan species. Like other mollusks, *pao/BEL* in *R. philippinarum* is also identified as the second most abundant superfamily of LTR retrotransposons (Supplementary Table S2) [19]. Like retroviruses, LTR retrotransposons can move between organisms and integrate new copies or new insertions into new host genomes by horizontal transfer events [88,89,90]. Recently, horizontal transfer of retrotransposons has been indicated among bivalves and other aquatic species of multiple phyla, whereas *pao/BEL* elements do not occur in mammals [77]. Together with the present study, the widespread occurrence of *pao/BEL* in molluscan genomes supports the hypothesis that *pao/BEL* elements may have evolved during early metazoan evolution [19,90]. It is therefore suggested that the remarkable expansion of *pao/BEL* in clam *R. philippinarum* may play an important role in genome evolution of the buried bivalves.

**Figure 6:**
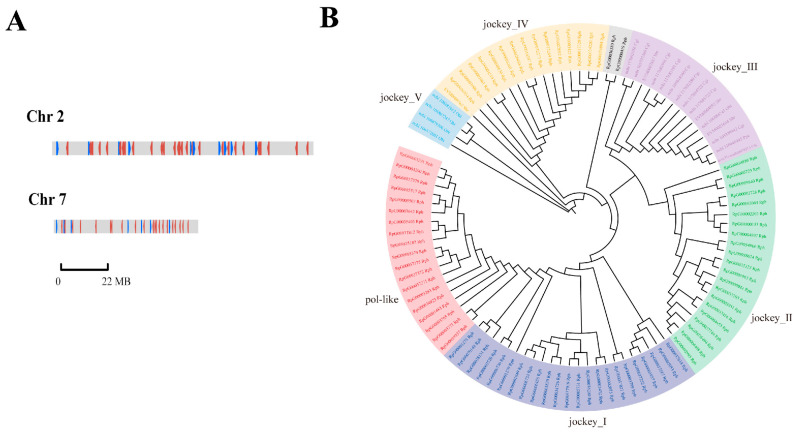
The chromosomal location and significant expansion of non-LTR retrotransposon elements in clam *R. philippinarum*. (**A**) The location of non-LTR retrotransposons in chromosomes 2 and 7; (**B**) Phylogenetic tree of non-LTR retrotransposons (*pol-like*, *jockey*_I, *jockey*_II, and *jockey*_IV) in Rph (*R. philippinarum*), Cgi (*C. gigas*), Pye (*P. yessoensis*), Cfa (*Chlamys farreri*), Sbr (*Scapharca broughtonii*) and *Obi* (*Octopus bimaculoides*).

Most non-LTR retrotransposons were located in chromosomes 2 and 7, oriented in both directions (Figure 6A). As illustrated in Figure 6B, six clades of non-LTR elements were revealed by analysis of gene family expansions and contractions. Significant expansion of non-LTR elements in *R. philippinarum* was identified as 80 non-LTR retrotransposons forming into four large clades (*pol-like*, *jockey*_I, *jockey*_II, and *jockey*_IV). Most notably, the three clades (*pol-like*, *jockey_1* and *jockey_2*) were recognized as the unique non-LTR elements in the clam *R. philippinarum*. In contrast, *jockey_3* was mainly detected in oysters and scallops, whereas *jockey_5* was only found in octopus. The contribution of transposable elements to genome sizes has been indicated in many vertebrates, especially in fishes [74]. Like fishes, mollusks show a wide range of genome sizes, ranging from 0.34 Gb in owl limpet *Lottia gigantea*) to 2.7 Gb in California two-spot octopus *O. bimaculoides* [27,52]. In the present study, the genome size of Manila clam was estimated to be 1.17 G, comparable to that of razor clam (1.22 G), but it was larger than that of Pacific oyster (0.55 G) and Zhikong scallop (0.76 G) [7,51,91]. Recently, the contribution of transposable elements to genome sizes has been evidenced by different genome sizes of two mussels, the deep-sea mussel *Bathymodiolus platifrons* (1.64 G) and the shallow-water mussel *Modiolus philippinarum* (2.38 G) [92]. These findings may imply that the variability in non-LTR retrotransposons may contribute to molluscan genome sizes, as well as their genome evolution.

**Figure 7:**
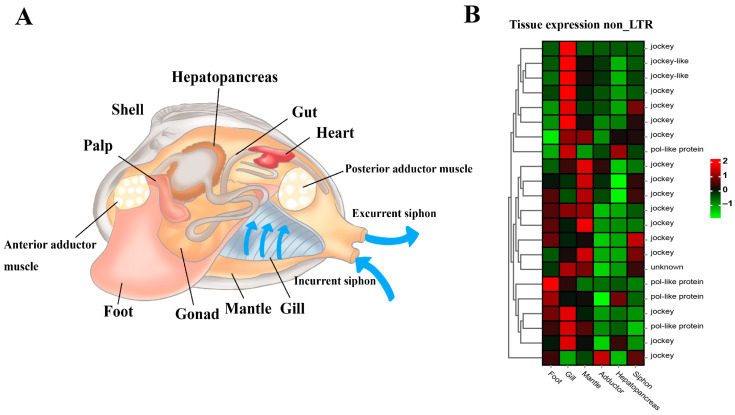
The general anatomy and tissue-specific expression of non-LTR transposable elements in the clam *R. philippinarum*. (**A**) The soft body structure of the clam, having foot, siphons, mantle, gill, adductor muscles, gonad and hepatopancreas; (**B**) The heatmap for tissue-specific expression of non-LTR retrotransposons. According to the normalized z-score FPKM values, the mean level of relative expression was calculated for each tissue using three biological replicates. The scale at the top right denoted normalized expression levels (red, high expression; blue, low expression). The heatmap for non-LTR retrotransposon expression was constructed by the normalized z-score FPKM values from tissue transcriptomic data.

The general anatomy indicates the soft body structure of the clam *R. philippinarum*, showing foot, siphons, mantle, gill, adductor muscles, gonad and hepatopancreas tissues (Figure 7A). Furthermore, comparative transcriptomics among different tissues revealed the tissue-specific expression pattern of these non-LTR retrotransposons (Figure 7B). As illustrated, non-LTR elements were highly expressed in gill, mantle, and foot. This may affect local gene expression in the functional tissues so as to increase organismal fitness through positive and purifying selection at the organism level [75,93,94,95,96]. As drivers of genomic and biological diversity, the expansion of non-LTR retrotransposons in clam *R. philippinarum* may not only affect the genome and transcriptome evolution, but also facilitate adaptive responses to environmental challenges and trigger novel adaptive phenotypes.

Gene family analysis revealed the significant expansion of E3 ubiquitin ligase in the clam (Supplementary Figure S4A). A total of 48 copies of E3 ubiquitin ligase were identified in the clams, while only four copies were detected in other molluscan species. The significant expansion of E3 ubiquitin ligase was also detected in the hard clam *M. mercenaria* (Supplementary Table S4). According to the comparative transcriptomics, tissue-specific expression of E3 ubiquitin ligase showed high expression in gill and mantle tissues (Supplementary Figure S4B). As the rate-limiting enzyme, E3 ligase is the most critical enzyme in the ubiquitin-proteasome system, serving as an important mechanism for protein degradation in the cytosol and nucleus [97,98]. Recent studies have revealed that ubiquitin is required to recover the normal transport between the nucleus and cytoplasm in cultured human cells and to disassemble stress granules in recovering cells [99,100,101]. A similar function of protein ubiquitin has also been detected in mollusks, having the potential to help cells to recover from environmental stress, especially for the recovery of cellular activities after heat shock [6]. For instance, protein ubiquitination and stress protein synthesis simultaneously increase during recovery from heat shock and tidal emersion in mussels, suggesting an essential role of protein ubiquitination in heat-stressed individuals [102,103,104,105]. Since temperature has been recognized as the key determinant of species distribution patterns, the significant expansion of E3 ubiquitin ligase found in the genome of *R. philippinarum* may be associated with their different intertidal habitats across latitudes [6,28,66,106]. Compared with other intertidal bivalves (e.g., oysters and scallops), clams (e.g., *R. philippinarum* and *M. mercenaria*) may be more likely to recover from thermal stress through preventing irreversible protein denaturation by means of stress protein synthesis and protein ubiquitination, especially in a particular thermal habitat. The present findings provide new evidences for understanding species distribution and habitat adaptation of infaunal bivalves by genome-level comparisons.

**Figure 8:**
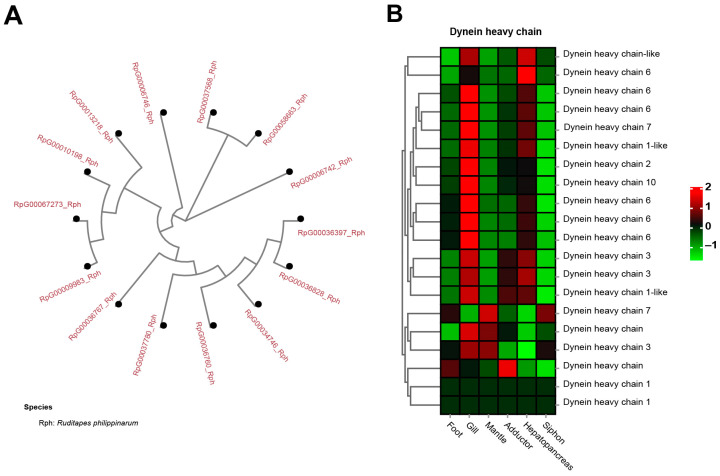
The significant expansion and tissue-specific expression of dynein heavy chain (DHC) in clam *R. philippinarum*. (**A**) phylogeny of the 13 copies of DHC in *R. philippinarum*; (**B**) the heatmap showing high expression of DHC in gill tissues. The mean relative expression level for each tissue was calculated using normalized z-score FPKM values in three replicates. The scale at the top right denoted normalized expression levels (red, high expression; blue, low expression).

Dynein heavy chain (DHC), a well-known molecular motor, was identified to be a significantly expanded gene family in the clams, having 13 copies of dynein heavy chain 3, containing AAA domains (Figure 8A). In contrast, none of them was found in the other five molluscan species. A heatmap of tissue-specific expression of DHC illustrated that gill showed relatively higher expression than did other tissues (Figure 8B). Dynein is one of the cytoskeletal molecular motors that can produce directed movement along microtubules [107,108]. For mollusks, dynein can stimulate the motility of neuronally-controlled gill cilia by cAMP-dependent protein phosphorylation [109]. Notably, the motor protein dynein heavy chain families were rapidly expanded in the razor clam *S. constricta* genome [91]. This is consistent with the significant expansion of DHC in the genome of clam *R. philippinarum*, suggesting their similar feature of burrowing behavior for adapting to the buried life. To adapt to infaunal life, bivalve mollusks can pump water for suspension-feeding by means of special cilia situated on the lateral parts of the filaments [110,111]. Gill pumping rates are associated with unit gill area, resulting in different feeding strategies in bivalve mollusks [110,111,112]. It is therefore suggested that gene family expansion of DHC might be part of an adaptative changes in size selection and particle-sorting filtration process in clams. For these burrowers adapting to the buried life, the capability of particle sorting and selection seems to be important to acquire food from the sandy seafloor.

### 3.4. Comparative Transcriptome Analysis and Immunohistochemical (IHC) Staining Revealed Tissue-Specific Expression of Extracellular Matrix (ECM)

Comparative transcriptome analysis revealed the tissue-specific gene expression in clams. In contrast to the adductor muscle, there were 570 up-regulated genes and 1321 down-regulated genes in the foot (Supplementary Figure S6). The greatest number of differentially expressed genes (DEGs) occurred between the gill and adductor, having 1326 up-regulated genes and 2468 down-regulated genes. The DEGs between siphon and adductor showed 686 up-regulated genes and 1431 down-regulated genes. These DEGs between pairwise tissues were enriched in a variety of pathways, such as calcium signaling pathway, ECM-receptor interaction, biosynthesis of amino acids, and metabolic pathways (Supplementary Figure S7). Co-expression gene networks were constructed by means of WGCNA, using 24 transcriptomes from eight adult tissues (Supplementary Figure S8A,B). Darkorange and darkorange2 were identified as the important modules significantly enriched in muscle-related tissues, such as the foot, mantle, and siphon (Supplementary Figure S8C,D). For these two modules, many genes were enriched in the pathways of extracellular matrix (ECM)-receptor interaction and neuroactive ligand-receptor interaction. 

**Figure 9:**
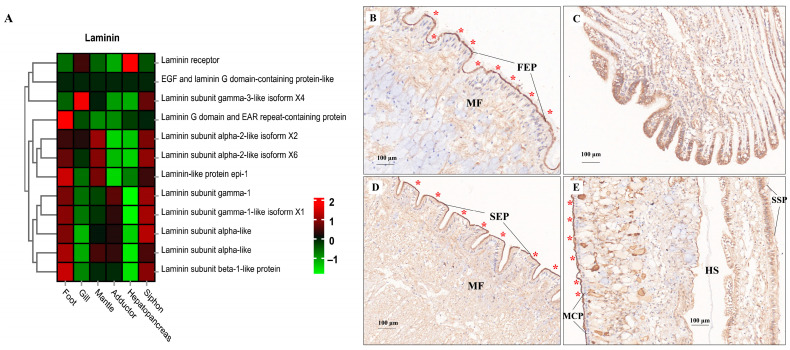
The mRNA heatmap and immunohistochemical (IHC) staining for laminin in different tissues of clams. The positive staining signals for laminin protein expression were indicated by the red asterisks. (**A**) mRNA expression heatmap for laminin among tissues; (**B**) laminin staining in foot showing the ciliated columnar epithelium (FEP), with the positive staining signals indicated by the red asterisks; (**C**) IHC for laminin indicating the skeletal bars of filaments (FB) in gills; (**D**) IHC for laminin in siphon epithelium (SEP); (**E**) IHC for laminin mantle tissue. MCP, mantle-cavity epithelium; SSP, shell-side epithelium; HS, hemolymph sinus; MF, muscle fibers. The mean relative expression level for each tissue was calculated using normalized z-score FPKM values in three replicates. The scale at the top right denoted normalized expression levels (red, high expression; blue, low expression).

**Figure 10:**
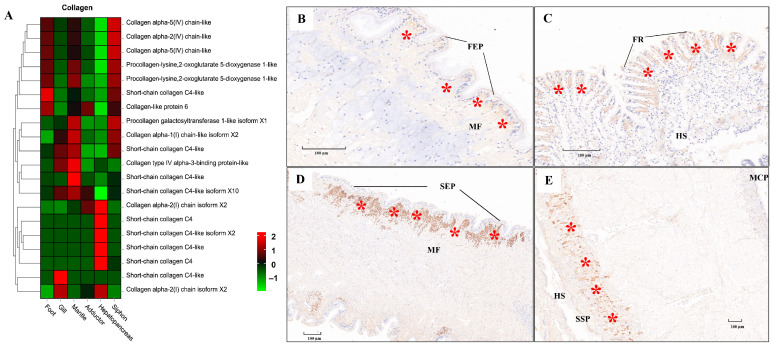
The mRNA heatmap and immunohistochemical (IHC) staining for collagen in different tissues of clams. The positive staining signals for collagen protein expression were indicated by the red asterisks. (**A**) mRNA expression heatmap for collagen among tissues; (**B**) collagen expressed under the foot epithelium (FEP), with the positive staining signals indicated by the red asterisks; (**C**) collagen detected under the frontal cilia (FR) of gills; (**D**) IHC for collagen in siphon epithelium (SEP); (**E**) IHC for collagen in mantle tissue. MCP, mantle-cavity epithelium; SSP, shell-side epithelium; HS, hemolymph sinus; MF, muscle fibers; FR, frontal cilia. The mean relative expression level for each tissue was calculated using normalized z-score FPKM values in three replicates. The scale at the top right denoted normalized expression levels (red, high expression; blue, low expression).

ECM components (e.g., laminin, collagen, and fibronectin) play fundamental roles in the structural integrity and biomechanical properties of different tissues [113,114,115]. In the present study, tissue-specific expression of laminin mRNA and protein was illustrated by the heatmap and IHC staining, respectively (Figure 9). Relatively higher mRNA expression of laminin was consistently found in muscle-related tissues (foot, siphon, and mantle) than in non-muscle tissues (gill and hepatopancreas) (Figure 9A). Laminin protein expression was mainly located in the epithelium (FEP) of the foot, siphon, and mantle tissues (Figure 9B–E). Consistently, laminin was found to be highly expression beneath the epithelium in many species of mollusks, serving as the conservative ECM components throughout the evolution of vertebrates and invertebrates [115]. Similarly, tissue-specific expression of collagen was also revealed by comparative transcriptomics (Figure 10A). The abundant mRNA expression of collagen was found in muscle-related tissues, especially in siphon (Figure 10). Notably, the high expression level of collagen was also supported by the IHC staining, which indicated the abundant expression of collagen under the siphon epithelium (SEP; Figure 10D). As previously indicated in scallops and mussels, collagen V was positively detected in the subepidermal tissues, while collagen VI was highly expressed in the mantle and adductor muscles, showing an intensely positive laminar structure [113,114,115]. In this study, the high expression of collagen VI was not only found in the mantle and adductor muscles, but also detected in the clam siphon. The similar expression pattern of collagen among mollusks reflects the functional role of collagen in basement membrane of the epidermal and muscle-related tissues in mollusks. Taken together, these findings provide the direct molecular and histological evidences that ECM components are serving the critical support for the basement membrane structure and composition in molluscan adult tissues, especially for their epidermal and muscle-related tissues. 

As previously reported, extracellular matrix (ECM) can not only provide structural support for tissues but also has a potential role in cell adhesion and mechanical characteristics [113]. More recent work highlights the key roles of ECM in the morphogenesis of neural tissues and animal behavior by controlling synapse structure and function [115,116,117]. For instance, ECM components can provide anchorage and mechanical buffering points for neurons and neurites and aid tissue morphogenesis [118]. For bivalve mollusks, ECM is also required for muscle and neuronal differentiation during primary cell culture, suggesting its important roles in tissue morphogenesis [115,119,120]. Furthermore, ECM receptors may modulate synaptic plasticity by cytoskeletal dynamics and synaptic activity, and thereby affect animal behavior [116]. For the clams, the wedge-shaped foot is well-adapted for burrowing into soft sediment, while siphons are responsible for respiratory seawater exchange and feeding behavior [23]. Clam siphons can be extended beyond the shell margin to a length greater than the shell length, contributing to adaptation to the buried beneath sediment, and thus enhancing survival (Figure 4). Therefore, the present findings of high ECM expression in clam muscle tissues may reflect its pivotal roles in tissue structure and function, especially for phenotypic adaptation to the buried lifestyle. 

**Figure 11:**
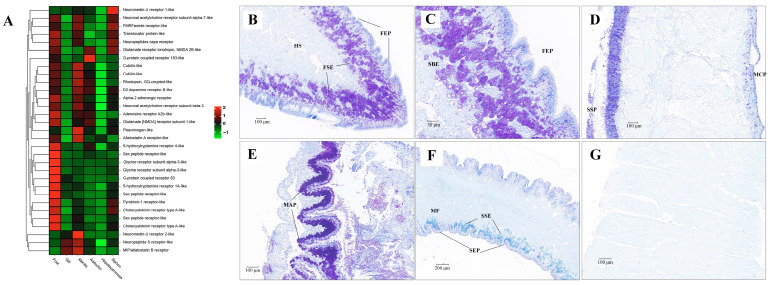
The mRNA heatmap and Nissl staining in different tissues of clams. (**A**) mRNA expression heatmap for neuroactive ligand among tissues; (**B**) abundant neuron cells identified in the foot sub-epithelium (FSE); (**C**) amplification of neuron cells in the foot sub-epithelium (FSE); (**D**) neuron cells detected in the shell-side epithelium (SSP) of mantle tissue; (**E**) neuron cells identified in the edge of major plica (MAP) of gill; (**F**) slight positive staining detected in siphon sub-epithelium (SSE); (**G**) No positive staining in the adductor muscle. MCP, mantle-cavity epithelium; SSP, shell-side epithelium; HS, hemolymph sinus; MF, muscle fibers. The mean relative expression level for each tissue was calculated using normalized z-score FPKM values in three replicates. The scale at the top right denoted normalized expression levels (red, high expression; blue, low expression).

Genes involved in the pathway of neuroactive ligand-receptor interaction were mainly expressed in foot, gill and siphon (Figure 11). Many kinds of neuroactive ligand-receptors were highly expressed in the foot, including neuromedin-U receptor, acetylcholine receptor, FMRFamide receptor, capa receptor, glutamate receptor, G-protein coupled receptor, dopamine receptor, adrenergic receptor, acetylcholine receptor, adenosine receptor, allatostatin-A receptor, 5-hydroxytryptamine receptor, glycine receptor, pyrokinin-1 receptor, cholecystokinin receptor, and neuropeptide S receptor. Furthermore, high expression of several of these neuroactive receptors was also found in siphons. In addition to neuroactive ligand-receptors, different isoforms of protocadherins (FAT1, FAT3, FAT4, and PCDH11X) were also found to be highly expressed in foot and siphon tissues of clams (Supplementary Figure S9). According to the Nissl staining in clam tissues (Figure 11), abundant neuron cells were mainly detected in foot sub-epithelium (FSE), shell-side mantle epithelium (SSP), gill major plica (MAP), and siphon sub-epithelium (SSE). In contrast, no positive staining in the adductor muscle was identified by the Nissl staining. As previously indicated, the flexible behavioral repertoires in mollusks are mainly controlled by the central nervous pathways [27,121,122]. For instance, foot contraction and extension were regulated by pedal ganglion, while the excurrent and incurrent siphons were innervated by siphonal ganglia [23,25]. The more recent work has confirmed a diverse repertoire of neurons in molluscan muscle tissues, suggesting that animal behavior is potentially regulated by the complex neural ganglia [20,27]. In the present study, high levels of neuroactive ligand-receptors expressed in foot and siphons are speculated to be involved in regulating body locomotion of clams (e.g., crawling, borrowing, and siphon extension) during their buried life. Furthermore, the significant expansion of gene families regarding to neuroactive ligand-receptor interaction in *R. philippinarum* and *M. mercenaria* may suggest the convergent evolution of enhanced nervous system for habitat adaptation in the infaunal bivalves. These findings shed light on the important roles of neural innervation in the generation of phenotypic adaptation in clams, supporting the claim of lineage-specific morphological novelties and “evo-devo” of mollusks [5].

## 4. Conclusions

We present a high-quality, chromosome-level genome assembly and cross-tissue transcriptome of the Manila clam *R. philippinarum*. The findings provide valuable molecular information for understanding the evolution of phenotypic adaptation to the buried life of some bivalves. Significant expansion of gene families were identified in the genome, including transposable elements, E3 ubiquitin ligase, dynein heavy chain and neuroactive ligand receptors. The findings of gene family expansion and tissue-specific expression may reflect the unique soft tissue structure of clams (e.g., wedge-shaped muscular foot and long extendible siphons) responsible for the buried lifestyle. The complex interplay of genomic architecture and gene functions most likely contributed to the evolution of lineage-specific morphological novelties in the infaunal bivalves.

## Figures and Tables

**Table 1:** Assembly and annotation statistics of Manila clam *Ruditapes philippinarum.*

Statistics/Species	This study	Xu et al., 2022 [31]	Yan et al., 2019 [18]	Mun et al., 2017 [29]
Sequencing strategy	PacBio + Illumina + Hi-C	PacBio + Illumina	Illumina	Illumina
Assembly level	Chromosome	Scaffold	Scaffold	Scaffold
Genome assembly statistics				
Assembly size (Gb)	1.17	1.41	1.32	1.37
Number of scaffolds	262		19	13318
N50 scaffold size (bp)	59,525,448		56,467,786	119,518
Number of contigs	5,371	15,908	61,395	121,896
N50 contig size (bp)	307,676	182,737	28,111	6,520
Genome features				
Protein-coding genes	37,428	34,505	27,652	108,034
Repeats (%)	54.17	48.20	38.29	26.38
GC (%)	32.11	32.00	31.89	
Genome quality assessment				
Complete BUSCOs (C)	902 (92.23%)	92.70%	92.20%	69.50%
Complete and single-copy BUSCOs (S)	763 (78.02%)	84.10%	90.30%	66.60%
Complete and duplicated BUSCOs (D)	139 (14.21%)	8.60%	1.90%	2.90%
Fragmented BUSCOs (F)	15 (1.53%)	2.60%	1.60%	11.20%
Missing BUSCOs (M)	61 (6.24%)	4.70%	6.20%	19.30%

## Data Availability

This genome project has been registered in the NCBI database under the BioProject accession PRJNA929581. The sequencing data have been deposited in the NCBI Sequence Read Archive (SRA) under the accession numbers SRR23279236, SRR23279237, and SRR23279238 for the genomic data. The BioProject accession for tissue transcriptomes has been registered as PRJNA928495, and the transcriptome data has been deposited under the accession numbers from SRR23249771 to SRR23249794.

## Supplementary Materials

The following supporting information can be downloaded at: https://www.mdpi.com/article/10.3390/biology13110870/s1, Table S1: The predicted non-coding RNA (ncRNA), including rRNA, tRNA, snRNA, and miRNA in the assembled genome of *R. philippinarum*; Table S2: The summary for all types of repetitive sequences detected in *R. philippinarum* genome; Table S3: The significant expansion of non-LTR retrotransposon, DNA transposons and LTR retrotransposon in *R. philippinarum* genome; Table S4: The summary for all types of repetitive sequences detected in *R. philippinarum* genome; Figure S1: The copy numbers of different types of repeat sequences in *R. philippinarum* genome; Figure S2: The venn diagram of the functional annotation by different databases (NR, SwissProt, GO, COG and KEGG); Figure S3: The venn diagram showing the common and unique gene families among the 13 invertebrates; Figure S4: The significant expansion and tissue-specific expression of E3 ubiquitin ligase in *R. philippinarum* genome; Figure S5: The GO and KEGG enriched analysis for gene family expansion in Manila clam *R. philippinarum* and the hard clam *Mercenaria mercenaria*; Figure S6: The statistics of differentially expressed genes (DEGs) revealed by comparative transcriptome analysis among different tissues of *R. philippinarum*; Figure S7: The enriched KEGG pathways for differentially expressed genes (DEGs) among different tissues of *R. philippinarum*; Figure S8: The results of WGCNA (weighted gene co-expression network analysis) for comparative transcriptomes among tissues; Figure S9: The different isoforms of protocadherins highly expressed in different tissues of *R. philippinarum*.
