# Supplementary material for: Clam Genome and Transcriptomes Provide Insights into Molecular Basis of Morphological Novelties and Adaptations in Mollusks
Source: Biology (Basel). 2024 Oct 25;13(11):870. doi: 10.3390/biology13110870 (PMC11592408; doi:10.3390/biology13110870)
Supplement: Supplementary file 1 [file biology-13-00870-s001.zip › biology-3251887-supplementary.pdf]

Supplementary Table S1. The predicted non-coding RNA (ncRNA), including rRNA, tRNA, snRNA, and miRNA in the assembled genome of *R. philippinarum*.

Type	Copy Number	Average Length(bp)	Total Length(bp)	Percentage of Genome
rRNA	17	114	1934	0.0002%
8s_rRNA	17	114	1934	0.0002%
tRNA	5963	73	436287	0.0371%
miRNA	367	105	38655	0.0033%
snRNA	171	143	24487	0.0021%
scaRNA	1	125	125	0.0000%
**Overall**	6536	674	503422	0.0429%

Supplementary Table S2. The summary for all types of repetitive sequences detected in *R. philippinarum* genome.

Class	Orders	Superfamily	Members	Length	Genomic proportion
DNA transposons	TIR	CMC	439	3881620	0.33%
DNA transposons	TIR	Ginger	27	141294	0.01%
DNA transposons	TIR	Harbinger	15	8387	0.00%
DNA transposons	TIR	MULE	293	331326	0.03%
DNA transposons	TIR	Merlin	19	2434	0.00%
DNA transposons	TIR	PIF	254	861641	0.07%
DNA transposons	TIR	PiggyBac	97	27191	0.00%
DNA transposons	TIR	TcMar	606	11592173	0.99%
DNA transposons	TIR	hAT	768	2702511	0.23%
DNA transposons	TIR	U	2	240	0.00%
DNA transposons	Crypton	Crypton	120	7145619	0.61%
DNA transposons	Helitron	Helitron	1932	355247153	30.23%
DNA transposons	Maverick	Maverick	78	2466683	0.21%
DNA transposons	MITE	MITE	499	46025186	3.92%
DNA transposons	Academ	Academ	50	524610	0.04%
DNA transposons	Dada	Dada	30	284797	0.02%
DNA transposons	IS3EU	IS3EU	31	4075568	0.35%
DNA transposons	Kolobok	Kolobok	73	432686	0.04%
DNA transposons	Novosib	Novosib	3	39274	0.00%
DNA transposons	Sola	Sola	101	520593	0.04%
DNA transposons	Zator	Zator	27	32266	0.00%
DNA transposons	Zisupton	Zisupton	2	162091	0.01%
DNA transposons	Unknow	Unknow	1	6379595	0.54%
LTR retrotransposons	LTR	P	71	88592	0.01%
LTR retrotransposons	LTR	Caulimovirus	20	3031	0.00%
LTR retrotransposons	LTR	Copia(Xen1)	2	108	0.00%
LTR retrotransposons	LTR	Copia	1450	417085	0.04%
LTR retrotransposons	LTR	ERV	4	899	0.00%
LTR retrotransposons	LTR	ERV1	427	618497	0.05%
LTR retrotransposons	LTR	ERV4	43	4500	0.00%
LTR retrotransposons	LTR	ERVK	283	399031	0.03%
LTR retrotransposons	LTR	ERVL	91	197209	0.02%
LTR retrotransposons	LTR	Gypsy	3022	33972915	2.89%
LTR retrotransposons	LTR	Unknow	90	64943	0.01%
LTR retrotransposons	LTR	Pao	566	2400677	0.20%
LTR retrotransposons	LTR	TATE	1	89	0.00%
LTR retrotransposons	LTR	Viper	1	81	0.00%
non-LTR retrotransposons	LINE	Ambal	4	3043	0.00%
non-LTR retrotransposons	LINE	CR1	444	8107904	0.69%
non-LTR retrotransposons	LINE	CRE	5	890	0.00%
non-LTR retrotransposons	LINE	DRE	7	14901	0.00%
non-LTR retrotransposons	LINE	Dong	6	3547	0.00%
non-LTR retrotransposons	LINE	Jockey	159	96673	0.01%
non-LTR retrotransposons	LINE	L1	767	8607526	0.73%
non-LTR retrotransposons	LINE	L2	630	36331305	3.09%
non-LTR retrotransposons	LINE	LINE	7	316371	0.03%
non-LTR retrotransposons	LINE	LOA	26	3406	0.00%
non-LTR retrotransposons	LINE	Penelope	120	2312987	0.20%
non-LTR retrotransposons	LINE	Proto1	5	8604	0.00%
non-LTR retrotransposons	LINE	Proto2	19	738377	0.06%
non-LTR retrotransposons	LINE	R1	60	204217	0.02%
non-LTR retrotransposons	LINE	R2	74	2108572	0.18%
non-LTR retrotransposons	LINE	RTE	338	12516431	1.07%
non-LTR retrotransposons	LINE	Rex	30	215266	0.02%
non-LTR retrotransposons	LINE	Tad1	74	12081	0.00%
non-LTR retrotransposons	LINE	Zorro	2	175	0.00%
non-LTR retrotransposons	DIRS	I	281	37060292	3.15%
non-LTR retrotransposons	DIRS	DIRS	136	1501676	0.13%
non-LTR retrotransposons	DIRS	Ngaro	52	1823756	0.16%
non-LTR retrotransposons	SINE	5S	6	160201	0.01%

non-LTR retrotransposons	SINE	7SL	2	5584	0.00%
non-LTR retrotransposons	SINE	Alu	3	225	0.00%
non-LTR retrotransposons	SINE	B2	3	184724	0.02%
non-LTR retrotransposons	SINE	B4	6	218461	0.02%
non-LTR retrotransposons	SINE	Core	1	54	0.00%
non-LTR retrotransposons	SINE	Dong	1	3547	0.00%
non-LTR retrotransposons	SINE	ID	6	933	0.00%
non-LTR retrotransposons	SINE	L2	1	36331305	3.09%
non-LTR retrotransposons	SINE	MIR	14	1662103	0.14%
non-LTR retrotransposons	SINE	SINE	12	2788566	0.24%
non-LTR retrotransposons	SINE	tRNA	29	176982	0.02%
Low_complexity	Low_complexity	Low_complexity	1	39873	0.00%
Satellite	Satellite	Satellite	5	984529	0.08%
Simple_repeat	Simple_repeat	Simple_repeat	1	995629	0.08%
Other	Other	Composite	1	51	0.000%
Other	Other	DNA_virus	1	9694	0.001%
Other	Other	Other	3	1626	0.000%
Other	Other	centromeric	1	49	0.000%
Other	Other	subtelomeric	1	43	0.000%
**Total**			**14882**	**636604774**	**54.17%**

Supplementary Table S3. The significant expansion of non-LTR retrotransposon, DNA transposons and LTR retrotransposon in *R. philippinarum* genome.

**Expanded TE family**	**TE classification**	**TE name**	Rph_clam	Cgi_oyster	Pye_scallop	Cfa_scallop	Sbr_ark shell	Obi_octopus
ORTHOMCL9	Non-LTR retrotransposon	*jockey-like*	40	8	0	0	3	1
ORTHOMCL42	Non-LTR retrotransposon	*jockey*	21	1	1	1	1	4
ORTHOMCL87	Non-LTR retrotransposon	*pol-like*	20	0	0	0	0	0
ORTHOMCL222	DNA transposons	*IS481*	14	0	0	0	0	0
ORTHOMCL433	LTR retrotransposon	*pao/BEL*	11	0	0	0	0	0

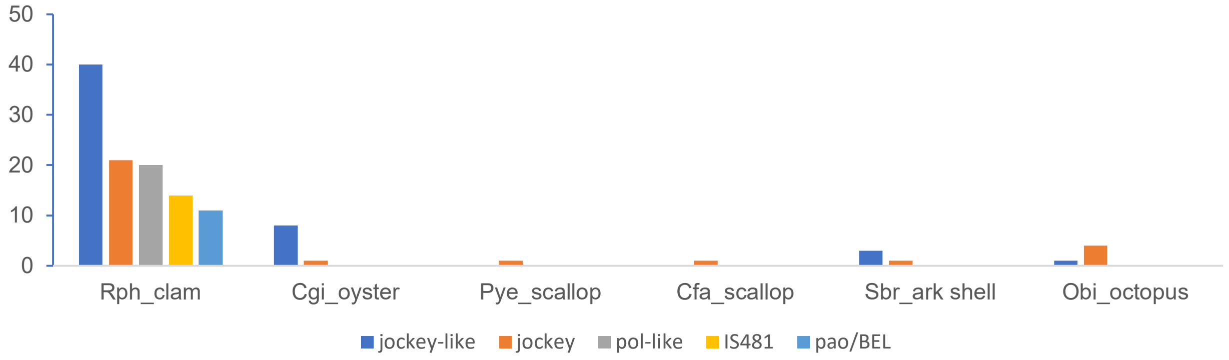

Supplementary Table S4. The summary for all types of repetitive sequences detected in *R. philippinarum* genome.

ORTHOMCL_id	ORTHOMCL_stat	Rph	Mme	Cfa	Pye	Cgi	Sbr	Obi	**Gene**
ORTHOMCL2	78 genes,5 taxa	64	0	0	4	6	3	1	RNA-directed DNA polymerase from mobile element jockey-like
ORTHOMCL3	71 genes,7 taxa	21	23	2	2	19	3	1	Fibrillin-1
ORTHOMCL9	48 genes,6 taxa	13	8	3	5	6	13	0	sacsin-like
ORTHOMCL10	46 genes,6 taxa	8	20	6	5	3	4	0	fusarin C synthetase-like
ORTHOMCL12	43 genes,6 taxa	11	4	6	3	8	11	0	Hemicentin-1
ORTHOMCL13	43 genes,6 taxa	5	17	3	4	10	4	0	E3 ubiquitin-protein ligase
ORTHOMCL19	39 genes,5 taxa	17	9	5	3	0	5	0	interferon-induced very large GTPase 1-like
ORTHOMCL22	33 genes,7 taxa	7	10	1	7	3	4	1	poly [ADP-ribose] polymerase 14-like
ORTHOMCL33	30 genes,7 taxa	22	1	1	1	2	1	2	RNA-directed DNA polymerase from mobile element jockey
ORTHOMCL37	29 genes,6 taxa	5	7	5	1	4	7	0	titin-like
ORTHOMCL46	25 genes,3 taxa	22	0	0	2	1	0	0	RNA-directed DNA polymerase from mobile element jockey-like
ORTHOMCL49	24 genes,6 taxa	17	1	1	3	1	1	0	neuroglobin-2-like
ORTHOMCL57	23 genes,2 taxa	20	3	0	0	0	0	0	tripartite motif-containing protein 2
ORTHOMCL62	22 genes,5 taxa	4	10	0	1	6	1	0	three-prime repair exonuclease 1
ORTHOMCL73	20 genes,6 taxa	1	11	2	1	1	4	0	zinc finger homeobox protein 4-like
ORTHOMCL77	20 genes,1 taxa	20	0	0	0	0	0	0	pol-like protein
ORTHOMCL78	19 genes,4 taxa	11	0	0	2	4	2	0	TPA_exp: polyprotein
ORTHOMCL87	19 genes,5 taxa	6	4	1	0	7	0	1	neuronal acetylcholine receptor subunit alpha-6-like
ORTHOMCL88	19 genes,2 taxa	18	1	0	0	0	0	0	probable E3 ubiquitin-protein ligase TRIM8
ORTHOMCL107	18 genes,6 taxa	12	1	1	2	1	1	0	fibrinogen C domain-containing protein 1-like
ORTHOMCL108	18 genes,4 taxa	13	2	1	2	0	0	0	transcription intermediary factor 1-alpha
ORTHOMCL126	17 genes,7 taxa	9	1	2	1	2	1	1	transmembrane protein 211-like
ORTHOMCL135	17 genes,2 taxa	16	1	0	0	0	0	0	--
ORTHOMCL136	17 genes,2 taxa	14	3	0	0	0	0	0	Dynein heavy chain 3, axonemal
ORTHOMCL137	17 genes,4 taxa	10	2	0	1	4	0	0	ryncolin-1
ORTHOMCL161	16 genes,7 taxa	1	10	1	1	1	1	1	methyltransferase-like protein 7A
ORTHOMCL165	16 genes,2 taxa	11	5	0	0	0	0	0	G-protein coupled receptor
ORTHOMCL166	16 genes,2 taxa	15	1	0	0	0	0	0	Cyclic GMP-AMP synthase
ORTHOMCL167	16 genes,2 taxa	15	1	0	0	0	0	0	Ankyrin repeat domain-containing protein 50

ORTHOMCL168	16 genes,2 taxa	13	0	0	0	3	0	0	C-type lectin domain family 4 member G-like
ORTHOMCL203	15 genes,2 taxa	14	1	0	0	0	0	0	tripartite motif-containing protein 2-like
ORTHOMCL204	15 genes,2 taxa	0	14	0	0	1	0	0	#N/A
ORTHOMCL227	14 genes,3 taxa	10	0	0	0	1	3	0	G2/M phase-specific E3 ubiquitin-protein ligase
ORTHOMCL256	14 genes,2 taxa	11	3	0	0	0	0	0	deleted in malignant brain tumors 1 protein-like
ORTHOMCL257	14 genes,2 taxa	12	0	0	0	0	0	2	NF-X1-type zinc finger protein NFXL1
ORTHOMCL258	14 genes,1 taxa	14	0	0	0	0	0	0	protein shisa-5-like
ORTHOMCL259	14 genes,1 taxa	14	0	0	0	0	0	0	Protein CBG07546
ORTHOMCL312	13 genes,3 taxa	10	2	0	0	0	1	0	T-cell-specific guanine nucleotide triphosphate-binding protein 1-like
ORTHOMCL339	13 genes,6 taxa	6	1	3	1	1	0	1	Pyroglutamylated RFamide peptide receptor
ORTHOMCL340	13 genes,3 taxa	11	1	0	0	1	0	0	hypothetical protein CAPTEDRAFT_203769
ORTHOMCL359	12 genes,3 taxa	10	0	0	0	1	1	0	RNA-directed DNA polymerase from mobile element jockey
ORTHOMCL415	12 genes,5 taxa	7	1	0	1	1	2	0	ATP-dependent DNA helicase Q-like SIM
ORTHOMCL428	12 genes,5 taxa	8	0	1	1	1	1	0	cellular retinoic acid-binding protein 2-like
ORTHOMCL430	12 genes,3 taxa	9	1	0	2	0	0	0	probable E3 ubiquitin-protein ligase TRIM8
ORTHOMCL431	12 genes,2 taxa	11	1	0	0	0	0	0	G-protein coupled receptor Mth2
ORTHOMCL433	12 genes,2 taxa	11	1	0	0	0	0	0	Protein MB21D2
ORTHOMCL434	12 genes,4 taxa	9	1	1	1	0	0	0	Techylectin-5B
ORTHOMCL435	12 genes,3 taxa	8	2	0	0	2	0	0	C-type lectin domain family 4 member M-like
ORTHOMCL436	12 genes,3 taxa	9	0	0	1	2	0	0	Integrase/recombinase xerD-like
ORTHOMCL437	12 genes,3 taxa	10	0	0	1	1	0	0	tigger transposable element-derived protein 1-like
ORTHOMCL489	11 genes,3 taxa	7	1	0	0	0	3	0	hypothetical protein LOTGIDRAFT_164522
ORTHOMCL526	11 genes,4 taxa	7	0	0	1	2	1	0	RNA-directed DNA polymerase from mobile element jockey
ORTHOMCL580	11 genes,2 taxa	10	1	0	0	0	0	0	probable G-protein coupled receptor 139
ORTHOMCL581	11 genes,2 taxa	10	1	0	0	0	0	0	probable E3 ubiquitin-protein ligase TRIM8
ORTHOMCL582	11 genes,2 taxa	10	1	0	0	0	0	0	G-protein coupled receptor GRL101
ORTHOMCL585	11 genes,2 taxa	10	1	0	0	0	0	0	NACHT, LRR and PYD domains-containing protein 3-like
ORTHOMCL588	11 genes,4 taxa	4	1	2	4	0	0	0	Neuronal acetylcholine receptor subunit alpha-6
ORTHOMCL590	11 genes,2 taxa	10	0	0	0	1	0	0	reverse transcriptase-like protein
ORTHOMCL591	11 genes,1 taxa	11	0	0	0	0	0	0	Gag-Pol polyprotein
ORTHOMCL592	11 genes,3 taxa	8	0	0	0	1	0	2	reverse transcriptase
ORTHOMCL756	10 genes,3 taxa	8	0	0	0	1	1	0	protein ALP1-like

ORTHOMCL782	10 genes,3 taxa	8	1	0	0	1	0	0	contactin-1-like
ORTHOMCL786	10 genes,2 taxa	9	0	0	0	0	0	1	histone-lysine N-methyltransferase SETMAR-like
ORTHOMCL787	10 genes,2 taxa	9	0	0	0	1	0	0	Jerky-like
ORTHOMCL1175	9 genes,1 taxa	9	0	0	0	0	0	0	RNA-directed DNA polymerase from transposon BS
ORTHOMCL1177	9 genes,2 taxa	8	0	0	0	1	0	0	pol-like protein
ORTHOMCL2211	8 genes,2 taxa	7	0	0	0	1	0	0	Endoplasmic reticulum resident protein 44
ORTHOMCL5583	7 genes,1 taxa	7	0	0	0	0	0	0	RNA-directed DNA polymerase from transposon BS
ORTHOMCL5585	7 genes,1 taxa	7	0	0	0	0	0	0	pol polyprotein-like protein
ORTHOMCL5586	7 genes,1 taxa	7	0	0	0	0	0	0	Retrovirus-related Pol polyprotein from transposon

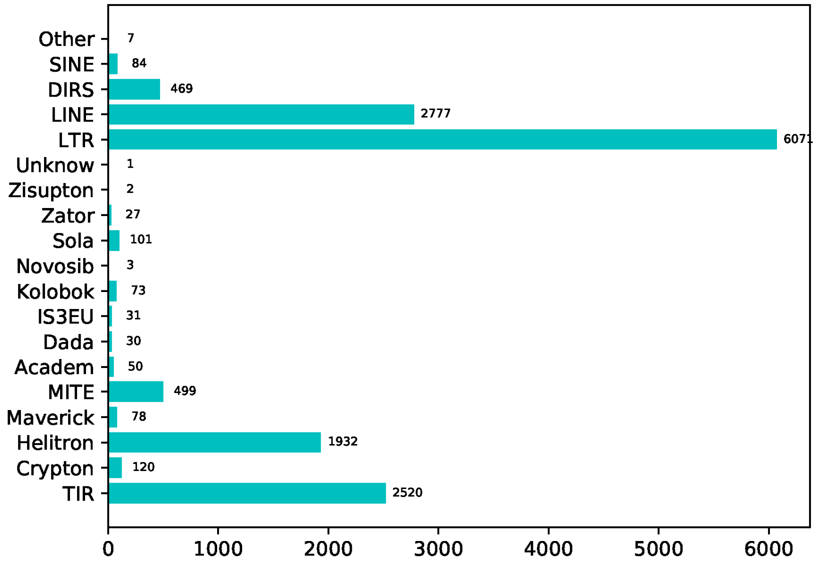

Supplementary Figure S1 The copy numbers of different types of repeat sequences in *R. philippinarum* genome.

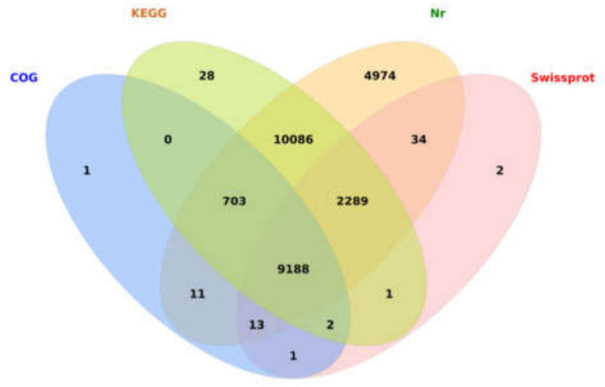

Supplementary Figure S2 The venn diagram of the functional annotation by different databases (NR, SwissProt, GO, COG and KEGG).

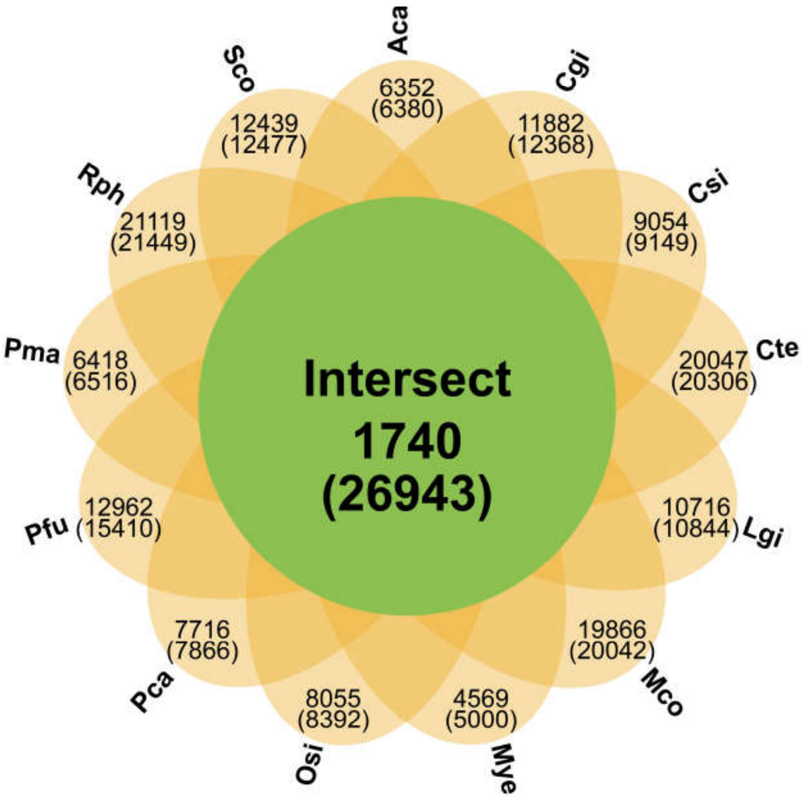

Supplementary Figure S3 The venn diagram showing the common and unique gene families among the 13 invertebrates, including Rph (*R. philippinarum*), Mco (*Mytilus coruscus*), Mye (*Mizuhopecten yessoensis*), Cgi (*Crassostrea gigas*), Csi (*Cyclina sinensis*), Sco (*Sinonovacula constricta*), Pma (*Pinctada fucata martensii*), Osi (*Octopus sinensis*), Lgi (*Lottia gigantean*), Pca (*Pomacea canaliculate*), Aca (*Aplysia californica*), Pma (*Pecten maximus*) and Cte (*Capitella teleta*).

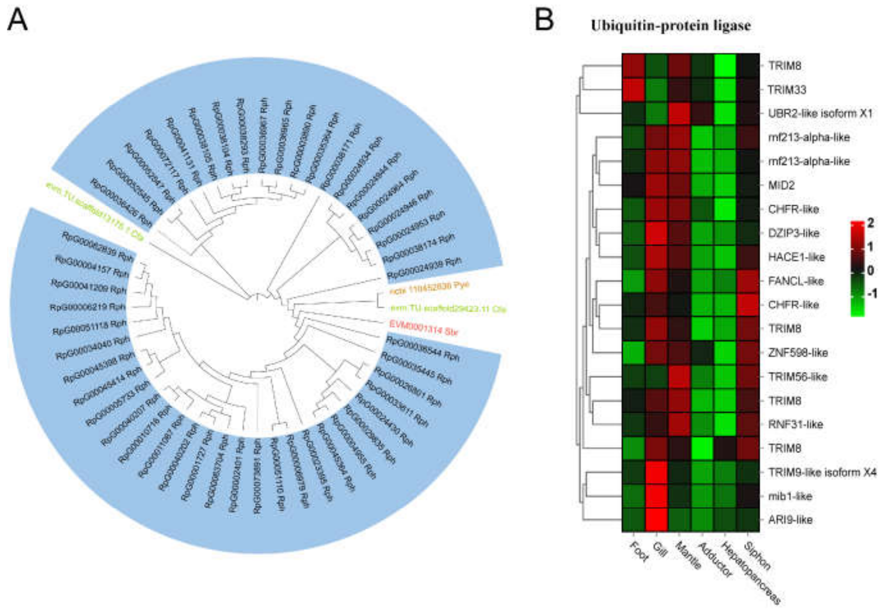

Supplementary Figure S4 The significant expansion and tissue-specific expression of E3 ubiquitin ligase in *R. philippinarum* genome. A) most of gene copies of E3 ubiquitin ligase expanded in *R. philippinarum* genome; B) the high expression levels of E3 ubiquitin ligase in gill and mantle tissues.

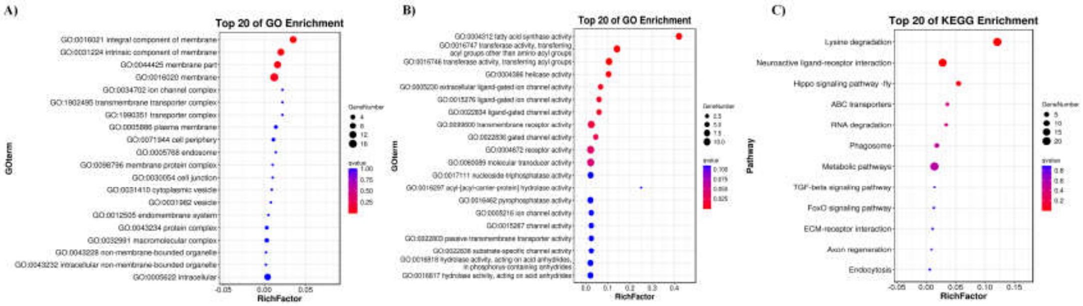

Supplementary Figure S5 The GO and KEGG enriched analysis for gene family expansion in Manila clam *R. philippinarum* and the hard clam *Mercenaria mercenaria.* A) top 20 of GO enrichment in cellular component; B) top 20 of GO enrichment in molecular function; C) top 20 of KEGG enrichment.

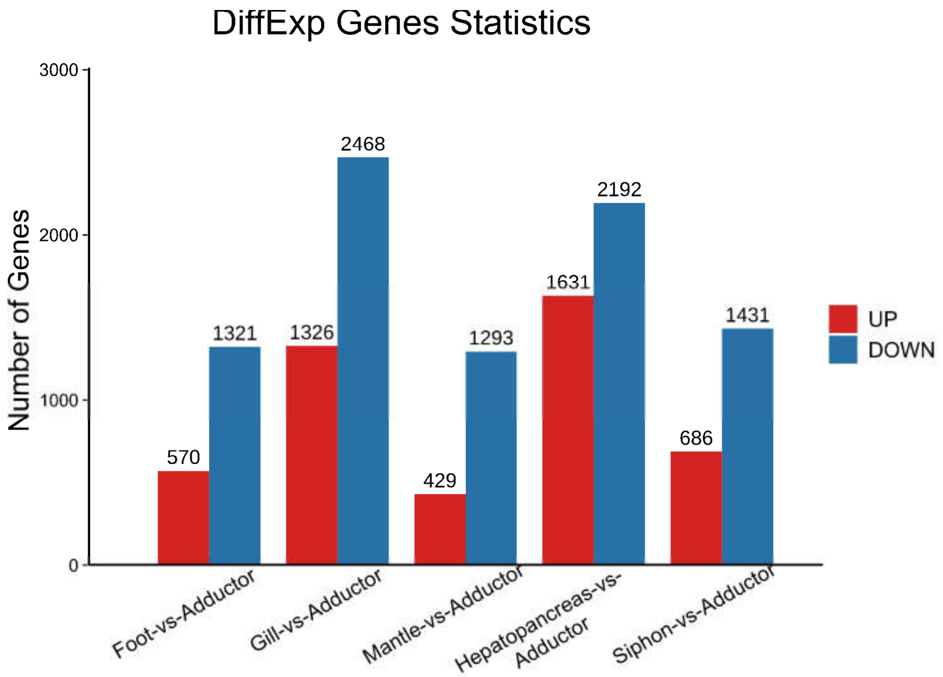

Supplementary Figure S6 The statistics of differentially expressed genes (DEGs) revealed by comparative transcriptome analysis among different tissues of *R. philippinarum*.

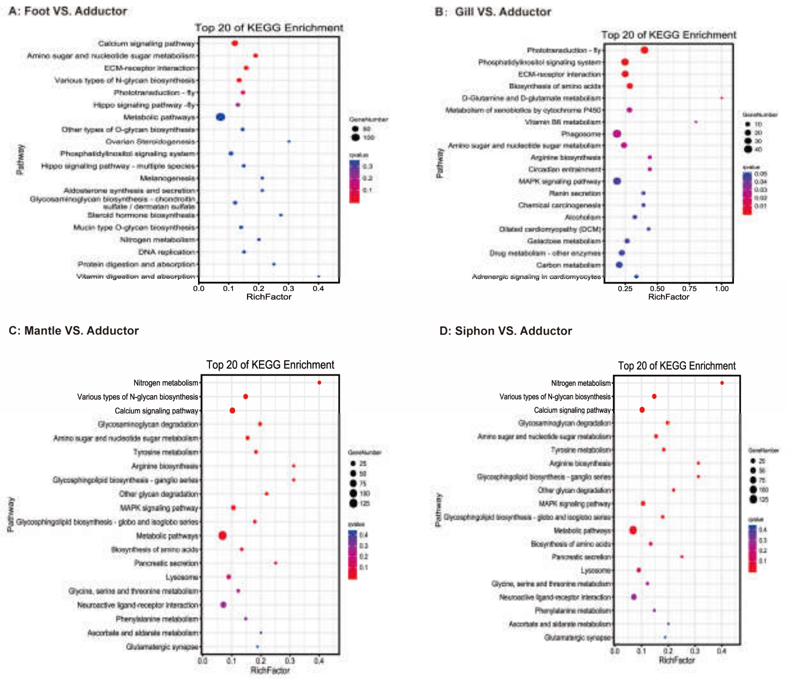

Supplementary Figure S7 The enriched KEGG pathways for differentially expressed genes (DEGs) among different tissues of *R. philippinarum*. A) foot VS. adductor; B) gill VS. adductor; C) mantle adductor; D) siphon adductor.

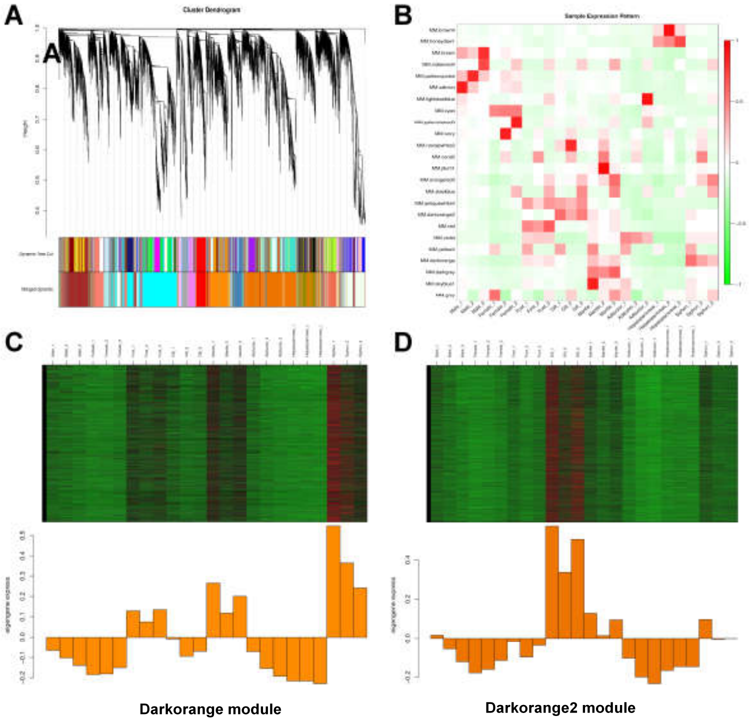

Supplementary Figure S8 The results of WGCNA (weighted gene co-expression network analysis) for comparative transcriptomes among tissues. A) the cluster diagram of the co-expression network constructed by 24 transcriptomes of eight tissues; B) the heatmap illustrating the expression pattern of samples; C) darkorange identified as the important module enriched in foot, mantle and siphon; D) darkorange2 identified as the important module enriched in gill and mantle.

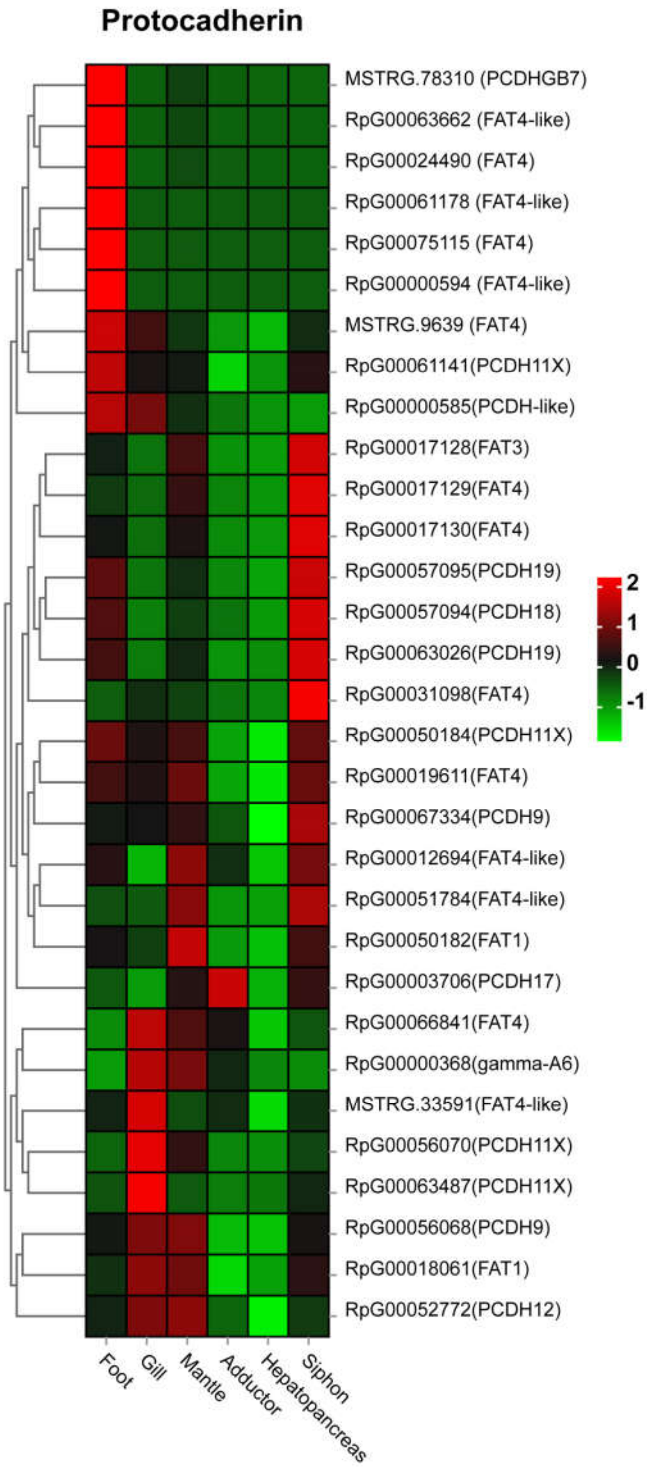

Supplementary Figure S9 The different isoforms of protocadherins highly expressed in different tissues of *R. philippinarum*.
